# Bibliometric study on clinical research of osteoporosis in adolescents

**DOI:** 10.3389/fpubh.2023.1041360

**Published:** 2023-02-22

**Authors:** Dingshuang Li, Jingxi Ou, Yang Zeng, Lei Hou, Yu Yuan, Zhiyuan Luo

**Affiliations:** ^1^Eighth Clinical Medical College of Guangzhou University of Chinese Medicine, Foshan, Guangdong, China; ^2^Acupuncture and Rehabilitation Clinical School of Guangzhou University of Traditional Chinese Medicine, Guangzhou, China; ^3^Department of Science and Education Section, Foshan Hospital of Traditional Chinese Medicine, Foshan, Guangdong, China

**Keywords:** adolescents osteoporosis, CiteSpace, VOSviewer, bibliometric, knowledge graphs

## Abstract

**Objective:**

Focusing on the theme of “osteoporosis-related research in adolescents,” a systematic visualization of the developmental lineage, current research status, hot spots, and trends of adolescent osteoporosis was conducted to provide a reference for subsequent related research, clinical diagnosis, and treatment.

**Method:**

The Web of Science core database was used as the data source to retrieve the relevant literature and the bibliometrics method. An online bibliometric platform, CiteSpace, and VOSviewer software were used to conduct co-occurrence analysis on the authors, scientific research institutions, national cooperation, keywords, and funding sources to draw the relevant knowledge map.

**Result:**

A total of 1,199 publications from the Web of Science core database were included in this study. The number of published adolescent osteoporosis (AOP) studies has shown an upward trend over the past 29 years, with the United States being the major contributor to the field with the highest number of publications (291, 24.3%) and the highest number of citations (12,186). The international collaboration map shows that the United States is the country most focused on international collaborative exchanges, with the closest collaboration between the United States and Canada. The most influential research institutions and authors are Children's Hospital and Rauch F. the United States is the primary funding source for this research area. Research hotspots were mainly focused on “bone density,” “osteoporosis,” and “children.”

**Conclusion:**

These knowledge maps review the research hotpots in adolescent osteoporosis research over time, analyze and summarize the research process over the past 29 years, and predict future research directions.

## Introduction

Osteoporosis (OP) is a disease of reduced bone strength and increased bone fragility due to reduced bone mass and damage to bone microarchitecture, which is insidious and prone to fracture and has been an active “disease of the elderly” (1). In recent years, due to globalized, convenient, intelligent environment, and changed lifestyles, especially since the Coronavirus disease 2019 (COVID-19) swept the world, long-term home isolation, outdoor sports, physical exercise plummeted, the sequelae of COVID-19, all of which make the problem of bone loss cannot be ignored (2). Adolescents are at a critical stage of growth and development, and the danger of abnormal bone loss is self-evident.

AOP was defined as children with ≥2 long bone fractures before age 10 years or ≥3 long bone fractures with Bone mineral density (BMD) *Z*-score < -2 before age 19 years; they were diagnosed with AOP if they had ≥1 vertebral fracture caused by a minor external force, such as cough, regardless of their BMD value (3). However, during clinical work, researchers found that the general public is unaware of AOP, and many clinicians are not sufficiently concerned about children with this disease. In addition, compared with multicenter, large sample size clinical studies on age-related osteoporosis, early studies related to AOP were mainly case reports and individual cases (4–6), and large studies related to AOP are still very limited, which means that there is still a lot of research space in this field.

CiteSpace and VOSviewer are techniques based on clustering technology to perform co-word analysis, co-citation analysis, literature coupling analysis, collaborative network co-occurrence, and keyword co-occurrence on the literature in the research area, which can show the direction and development history of the research field from multiple angles. However, to our knowledge, there are still no previous bibliometric studies reporting the AOP clinical research. Therefore, we attempted to use bibliometric analysis as the method, focusing on keyword analysis to evaluate the current research hotspot and predict the possible future research trend of AOP. In this study, we used a variety of software programs and online platforms to map the scientific knowledge of AOP-related research areas. The main objectives of the study were to (1) identify the major contributors to the field of AOP from 1994 to 2022, including authors, institutions, and countries; (2) analyze the research focus of each period and explore its development and evolutionary trends; (3) predict the future research frontiers in the field; (4) provide some new perspectives and ideas for subsequent research in AOP; and (5) call for more attention, especially from clinical physicians and researchers on this topic.

## Materials and methods

### Data source

The data for this study were obtained from Science Citation Index Expanded (SCI-Expanded), Web of Science Core Collection (WOSCC), which is an internationally recognized database that reflects the level of scientific research. It includes amounts of influential and high-quality journals worldwide. It is also one of the most frequently used databases in previous bibliometric studies.

### Retrieval process

**Figure 1:**
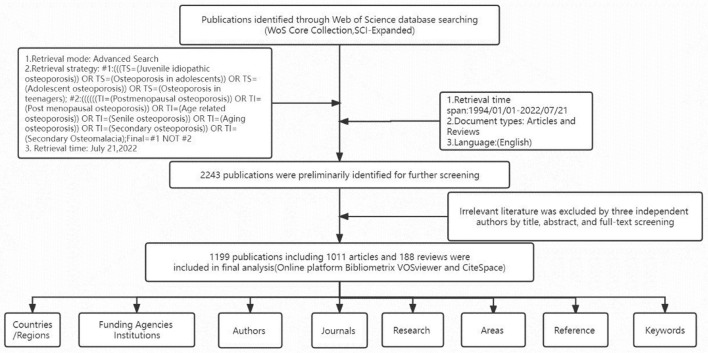
The specific data retrieval process of this study.

The search strategy was as follows (Figure 1). We selected publications identified through WOSCC in advanced retrieval mode. All potentially relevant publications were collected based on the topic (TS) and the title (TI) with the following search formula: #1: [(((TS=(Juvenile idiopathic osteoporosis) OR TS=(Osteoporosis in adolescents)) OR TS=(Adolescent osteoporosis)) OR TS=(Osteoporosis in teenagers)); #2:((((((TI=(Postmenopausal osteoporosis)) OR TI=(Post-menopausal osteoporosis)) OR TI=(Age-related osteoporosis)) OR TI=(Senile osteoporosis)) OR TI=(Aging osteoporosis)) OR TI=(Secondary osteoporosis)) OR TI=(Secondary Osteomalacia); Final dataset: #1 N #2], with Language choice English. Publication types were limited to articles and reviews, excluding papers in conference proceedings, letters, editorials, conference abstracts, news reports, editorials, corrections, early access, retracted publications, and non-English literature. Since the search was conducted on July 21, 2022, at 18:00, the results of this search covered publications between 1994 and 2022. The initial search results contained 2,243 published articles. After independent manual screening and discussion of the search results by three authors, 1,199 articles were included in this study, including 1,011 papers and 188 reviews. Finally, the 1,199 included studies were visually analyzed for publication country/region, grant program, author, journal, investigator, research area, citation, and keywords.

### Data filtering and export

In this study, the selection, input, and collection of data was done collaboratively by Dingshuang Li, Jingxi Ou, and Yang Zeng. Data cleaning was achieved through manual screening, with each of the three authors performing the initial classification and screening of 2,243 articles. The screening was performed by reading article abstracts, keywords, and full text. Only literature on clinical research topics related to AOP was included in this study, excluding all studies that were not on human subjects such as plant and animal studies and basic trials, only articles and reviews were included, and only English-language literature was included. However, during the screening process, we found that many of the articles mentioned adolescent osteoporosis in only a small part of the text, rather than the full text on the topic, so they were therefore excluded. After manual screening, 1,199 papers were eventually included. After the manual screening and de-duplication and exclusion of animal experiments, 1,199 papers were finally included, and the form of the included papers was selected as “full record with cited references” and exported in plain text format and tab-delimited format, respectively. The data is downloaded from WOSCC in the file types “Tab File” and “Plain Text File” the plain text file is named “download_ ^***^.txt,” and the plain text file is imported into the CiteSpace software for analysis with the online bibliometrics website, and the tab file is imported into the VOSviewer software for analysis with the online bibliometric website. Three authors collaborated to perform screening and extraction data entry and collection jointly. The selected data included general information such as annual publication volume, country, distribution, funding agency, journal, author, reference, citation frequency, average citations per item (ACI), and H-Index.

### Bibliometric and visualized analysis

The tools used in this bibliometrics study include CiteSpace software developed by Professor Chaomei Chen, VOSviewer software developed by Drexel University in the United States, and two other online analysis platforms.

CiteSpace.5.7.R5 software was selected, Time Slice was set to 1 year, Selection Criteria was established as g-index and set *k* = 25, and LLR and Time-zone algorithms were applied to construct keyword clustering maps highlighting time zone maps and co-occurrence maps. CiteSpace is a visual analytics software that can analyze trends and dynamics in the scientific research literature and identify critical points in specific domains (7). Betweenness Centrality (B.C.) is an essential parameter for measuring the scientific importance of nodes in a network. The core research institutions and highly cited authors in the visualization graph drawn by CiteSpace software are both high (≥0.1) (8).

For the two-layer map coverage of journals, each label represents a different research topic covered by the relevant bulletin. The left side of the map shows the cited journals, and the right side shows the mentioned journals. Lines of different colors and widths from the citation map's beginning to the end indicate the path of citation links.

In this study, CiteSpace and VOSviewer used default parameters. Van Eck and Waltman developed VOSviewer at the Center for Science and Technology Studies (CWTS), Leiden University, The Netherlands. It is software for visualizing knowledge units of literature based on Visualization of Similarity (VOS) technology with a unique knowledge domain mapping display. VOSviewer is used to visualize co-authorship of countries, authors, and organizations, co-referencing resources, and co-occurrence of keywords. In the network visualization map created by VOSviewer, different nodes represent different parameters, such as countries, journals, and keywords. The size of the nodes in the mapping is proportional to the number of publications, citations, or occurrences. Total Link Strength (TLS) indicates the strength of the connection between a node and other nodes.

## Results

### Analysis of the outputs and trends of publications

**Figure 2:**
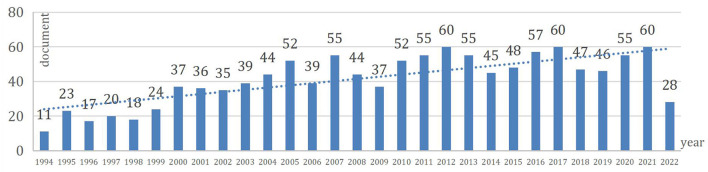
The amount of annual publications regarding AOP from 1994 to 2022.

Based on the literature search and screening strategy in Figure 1, 1,199 literature studies were finally identified, including 1,066 articles and 133 reviews. Figure 2 depicts the specific amount of annual publications regarding AOP. From 1994 to 2022, the number of published studies on osteoporosis in adolescents exhibited an upward trend, and the average growth rate of publications was 3.12%. Since the annual number of publications in the field exceeded 35 for the first time in 2000, the annual scientific research output has remained above 35, reaching 60 in 2012, 2017, and 2021. It is foreseeable that research in this area will continue to grow.

### Analysis of publications countries/regions

**Figure 3:**
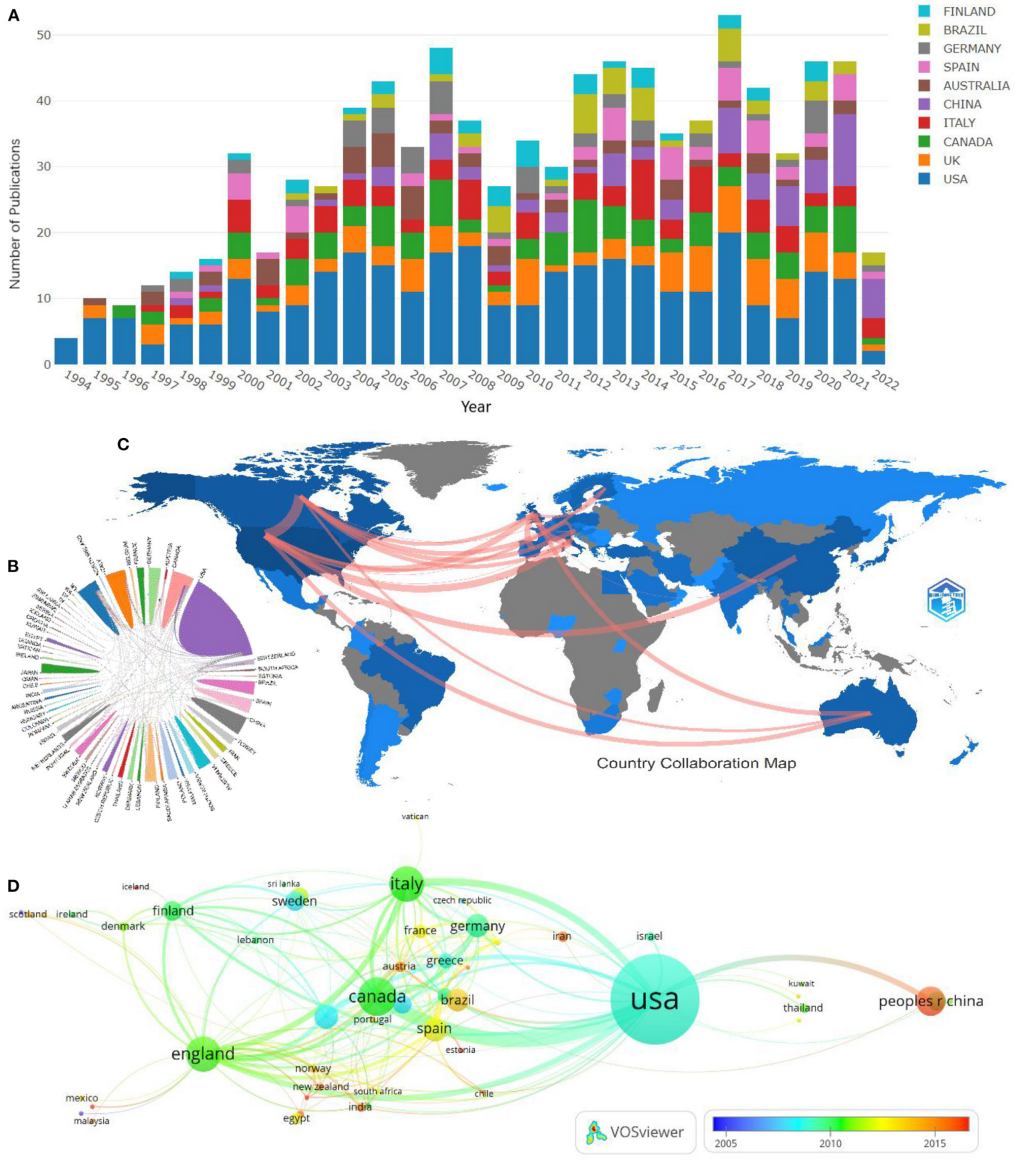
The top 10 countries in terms of annual publication volume **(A)**. Map depicting the international collaboration analysis among different countries **(B, C)**. Map of country co-authorship analysis **(D)**.

A total of 55 countries/regions contributed to all publications on AOP research, except for 22 articles whose provenance could not be identified. The top 10 countries/areas of publication of these papers are noted in Figure 3A, whose research accounted for 64.1% of the total publications. The annual output of 10 countries with high scientific production, led mainly by the United States, is shown in different colors. The USA was the only productive country, with 291 papers published (24.3%), followed by Canada (80, 6.7%) and Italy (74, 6.2%). Research from the USA was cited 12,186 times, ranked first of all the countries, followed by Canada (3,073 times) and Italy (2,889 times). The geographic distribution of cooperation across countries is shown on a map in Figure 3C, while in Figure 3B, line thickness between countries displays the intensity of the closeness. As shown in Figures 3B–D the USA and Canada get the most international cooperation.

### Analysis of institutional output

**Figure 4:**
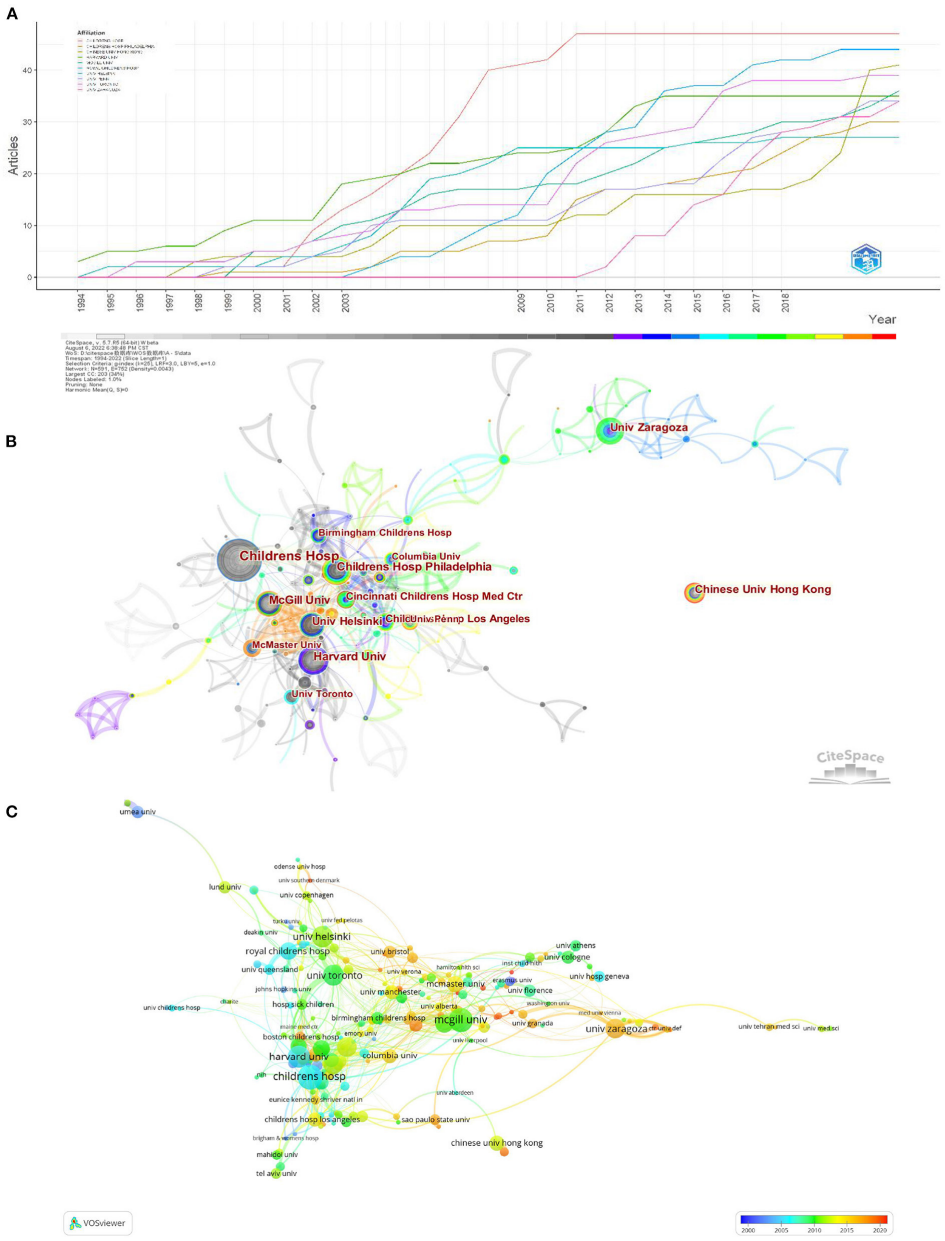
The amount of annual publications regarding AOP on top 10 institutions **(A)**; the research network on AOP by CiteSpace **(B)** and VOSview **(C)**.

For the analysis of institutions, 591 institutions made contributions to this field. The line chart of Figure 4A details the literature counts of the top 10 most productive institutions. Among these, the institutions with the most publications on AOP were the Children's Hospital (54 documents), followed by the University of Helsinki (44 documents), and the Chinese University of Hong Kong (39 papers).

Figures 4B, C generate collaborative visualization maps of the AOP research network by CiteSpace and VOSview. The circle nodes in both figures represent different institutions, the size of the nodes reflects the number of studies, and the connecting lines between each node in the machine reflect the intensity of cooperation between institutions. Institutions such as Children's Hosp, Univ Helsinki, and Harvard Univ as the center radiating outward constitute the AOP research network, indicating that these institutions pay attention to scientific research in the field of AOP while also focusing on communication and cooperation. However, judging from the overall cooperation network graph (all institutions have B.C. values < 0.1), although these institutions produce many research results, there is not enough communication among them. Inter-university collaboration is low and is mainly concentrated in European and American research hospitals. In addition, there is a relative lack of cooperation between institutions in different countries.

### Analysis of the most active funding agencies

**Figure 5:**
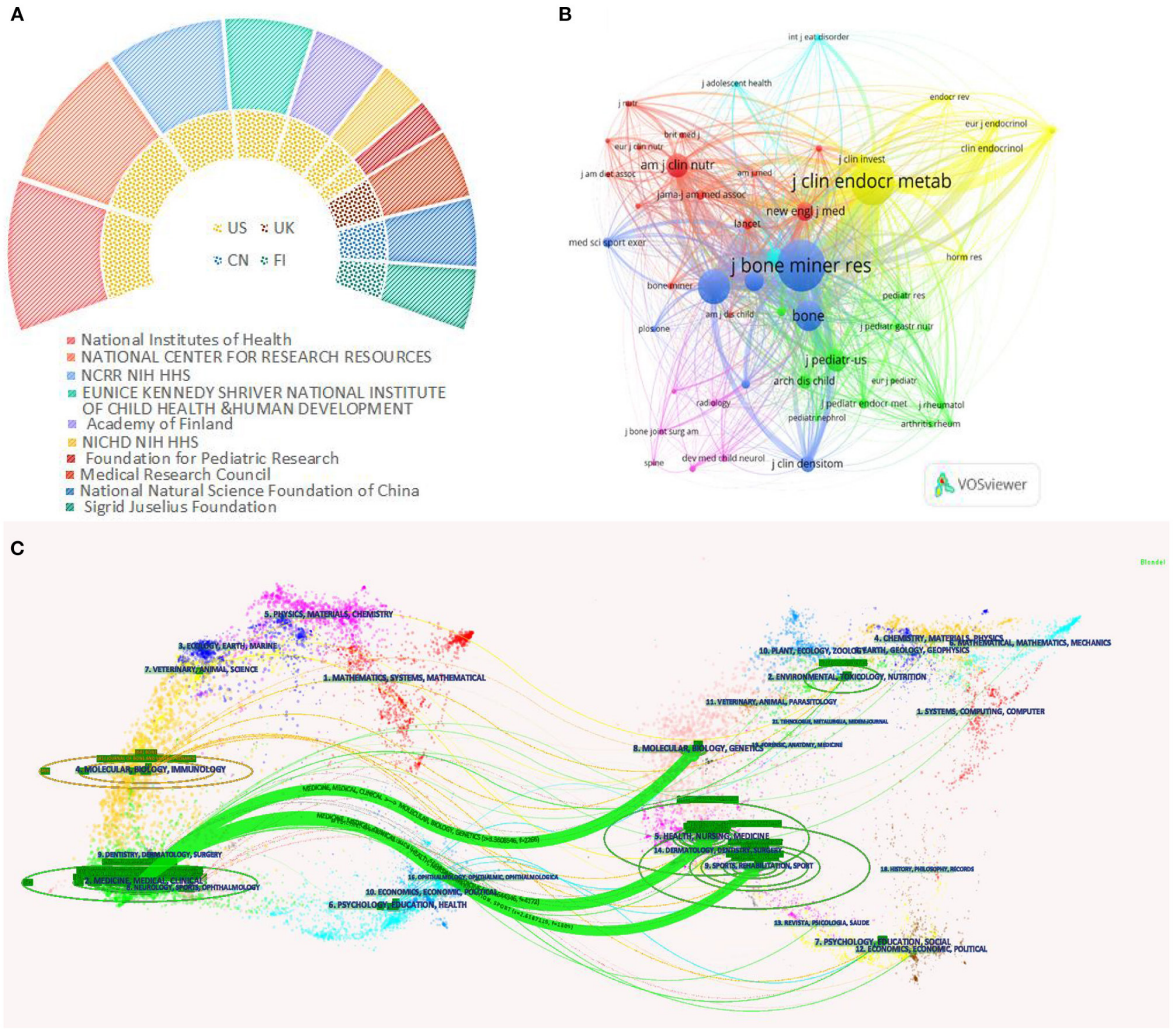
The top 10 most frequent funding sources on AOP **(A)**. Network visualization map of journal co-citation analysis **(B)**. The dual-map overlay of academic journals on AOP (generated by CiteSpace software) **(C)**.

Figure 5A summarizes the data of the top 10 most frequent funding sources in this field, with seven funding agencies based in the United States. The remaining were from China, Finland, and England. The top three most active funding agencies were the National Institutes of Health (NIH), USA (153 studies), National Institute Of Diabetes And Digestive And Kidney Diseases (161 studies), and Eunice Kennedy Shriver National Institute Of Child Health & Human Development (110 studies). It is clear from these results that the United States is well-positioned to create a more complete research body in the field of AOP than other countries, thanks to significant financial support. Canada also has more research but is underfunded. In addition, China and the U.K. each have a funding agency on the list, reflecting the importance that both countries place on research in the AOP field.

### Analysis of journals and co-cited journals

A total of 402 journals are included in the research area of AOP. As is shown in Figure 5B, VOSviewer is used to further perform the network visualization diagram of journal co-citation analysis. Only the top 50 journals which had been cited more than 150 times were shown in the picture. Table 1 lists the specific number of outputs of the 10 most published journals in the field and their 2020 Journal Impact Factor values and Journal Citation Indicator quartile. A dual-map overlay of the journals on AOP is shown in Figure 5C. On the left is the map of the citation, and on the right is the map of the cited. The curve between the left and right parts of the ellipse shows the complete citation process. In the left map, the more papers published in the journal, the longer the vertical axis of the ellipse. The greater the number of authors, the longer the horizontal axis of the ellipse.

**Table 1:** Top 10 Journals in terms of publication volume on AOP.

**Rank**	**Sources title**	**Output**	**% of 1,199**	**JIF (2020)**	**JIF (2021)**	**JCR (2020)**
1	Osteoporosis International	76	6	4.507	5.071	Q2
2	Bone	68	6	4.398	4.626	Q2
3	Journal of Bone and Mineral Research	45	4	6.741	6.39	Q1
4	Journal of Pediatric Endocrinology and Metabolism	43	4	1.674	1.52	Q3
5	Journal of Clinical Endocrinology and Metabolism	36	3	5.958	6.134	Q1
6	Journal of Clinical Densitometry	28	2	2.617	2.963	Q3
7	Calcified Tissue International	27	2	4.333	4	Q2
8	Journal of Bone and Mineral Metabolism	27	2	2.626	2.976	Q2
9	Journal of Pediatrics	21	2	4.406	6.314	Q1
10	Journal of Pediatric Gastroenterology and Nutrition	17	1	2.839	3.288	Q2

### Analysis of authors and co-cited authors

**Figure 6:**
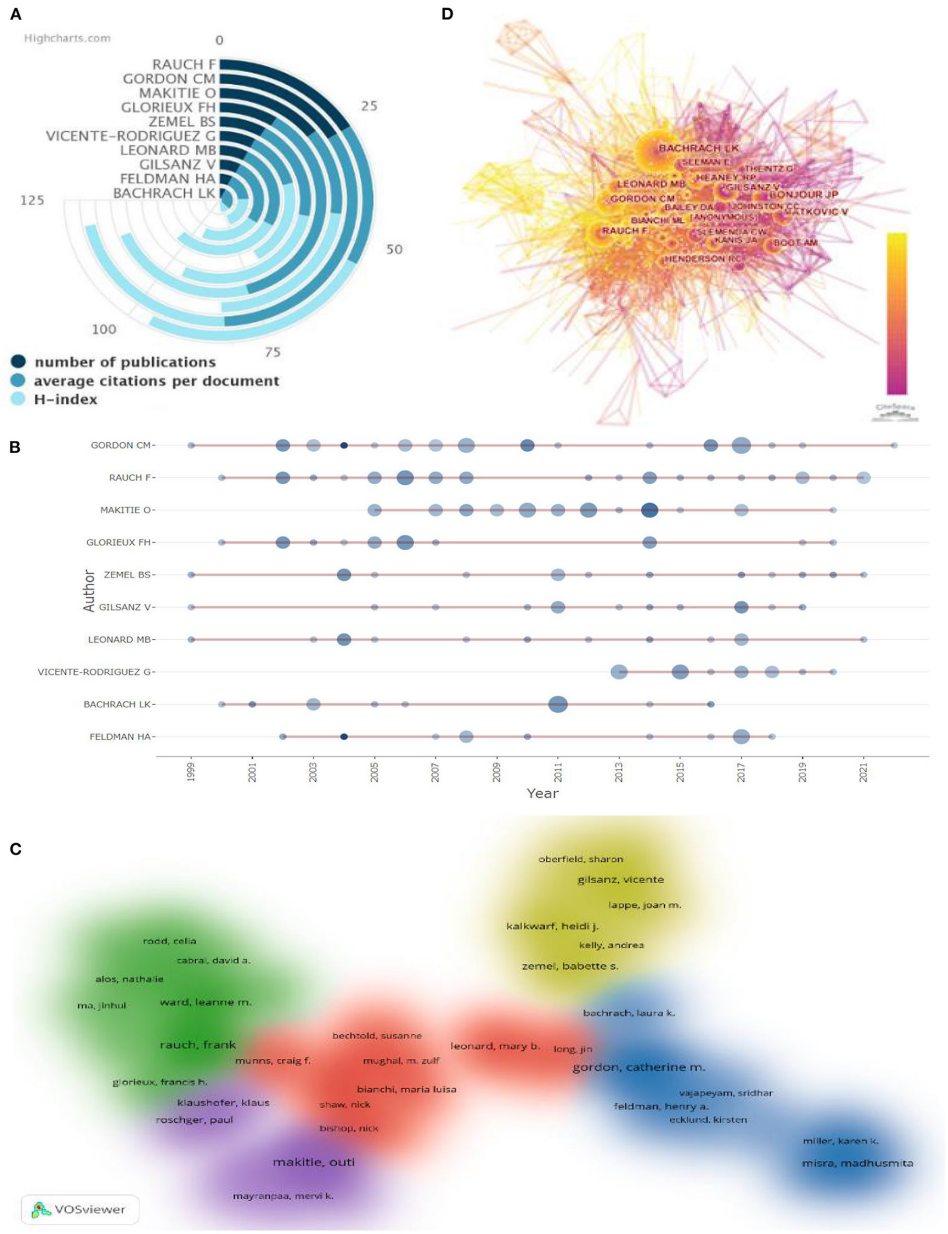
This area has a total number of publications, average citations per documents, and the H-index of the top 10 authors **(A)**. Top 10 authors' production over time **(B)**. Author co-authorship analysis **(C)**. Map of cited-author **(D)**.

The number of papers published by a researcher represents their contribution and activity level in the field. Figure 6A illustrates the top 10 researchers in total publications, where the number of publications, ACI, and IN-D are indicated in three shades of blue from dark to light, respectively. As shown in Figure 6A, among the top 10 most productive authors, Rauch F. (27;27.19;42) from McGill University and Catherine M. Gordon (27;55.45;37) from Baylor College of Medicine contributed the most articles, followed by Outi Mäkitie from University of Helsinki. Moreover, Francis H. Glorieux (15; 61.06;39) ranked first in ACI, while Mary B. Leonard (13;41.53;50) ranked first in the H-index. In Figure 6B, the circle's size indicates the author's number of publications in that year, while the shade of the color signifies the total number of citations. Taking Rauch F. as an example (27; 27.19; 42), since the publication of the author's first paper on AOP in 2000, the scientific output has been sustained from 2002 to 2008 and 2012 to 2022, with the effects of intravenous pamidronate on the bone tissue of children and adolescents with osteogenesis imperfecta being cited most frequently (TC = 226; TcpY = 10.762) (9). Figure 6C reveals the mutual collaboration among the authors. They created several research clusters, each from one or two core authors, such as Rauch and Outi Mäkitie, with relatively close ties within collections and less collaboration between different groups of researchers. CiteSpace represents nodes with centrality >0.1 as circles; the darker the color is, the more recent the year of citation; the more influential the node is, the more frequent the article has been cited. As shown in Figure 6D, Bachrach, Bonjour, and Gordon are the key authors in the co-citation network of the AOP field, and these people have influence.

### Analysis of keywords

**Figure 7:**
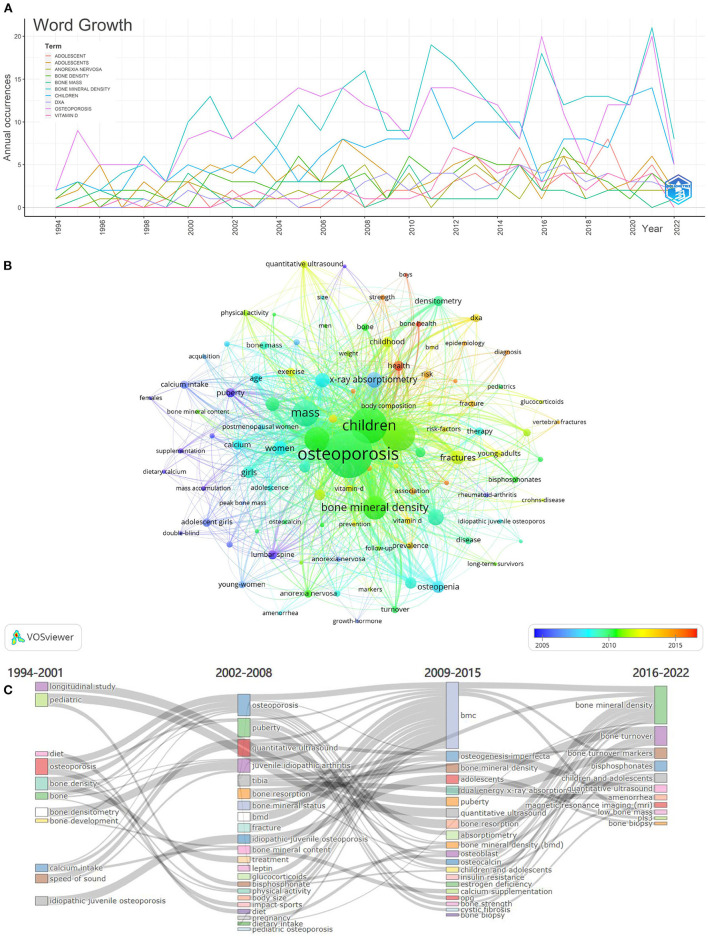
The annual number of the top 10 most frequent keywords in the AOP from 1994 to 2022 **(A)**. The time-overlay visualization map of the co-occurrence keywords was generated by using VOSviewer **(B)**. The keyword Sankey evolution diagram **(C)**.

Figure 7A shows the annual frequency distribution of the top 10 most frequently occurring keywords. The top five keywords were: bone mineral density (303 times), osteoporosis (297 times), children (193 times), adolescents (108 times), and bone density (91 times). As shown in Figure 7B, a density visualization map was generated for keywords with a co-occurrence more significant than 23 times, which includes 101 keywords in the map. These keywords were labeled with different colors, and an overlay visualization map was created to reflect the research hotspots in different periods. In addition, Bibliometrix was used for thematic evolution analysis. Figure 7C uses a Sankey diagram to explain the four phases of thematic evolution in the AOP study, showing the pattern of change in the annual frequency of author keywords related to adolescent osteoporosis from 1994 to 2022. The line between nodes reflects the research topic's evolving focus. The width indicates the number of shared keywords. The thicker the line, the greater the significance of the two subjects.

### Analysis of references and citation burst

**Figure 8:**
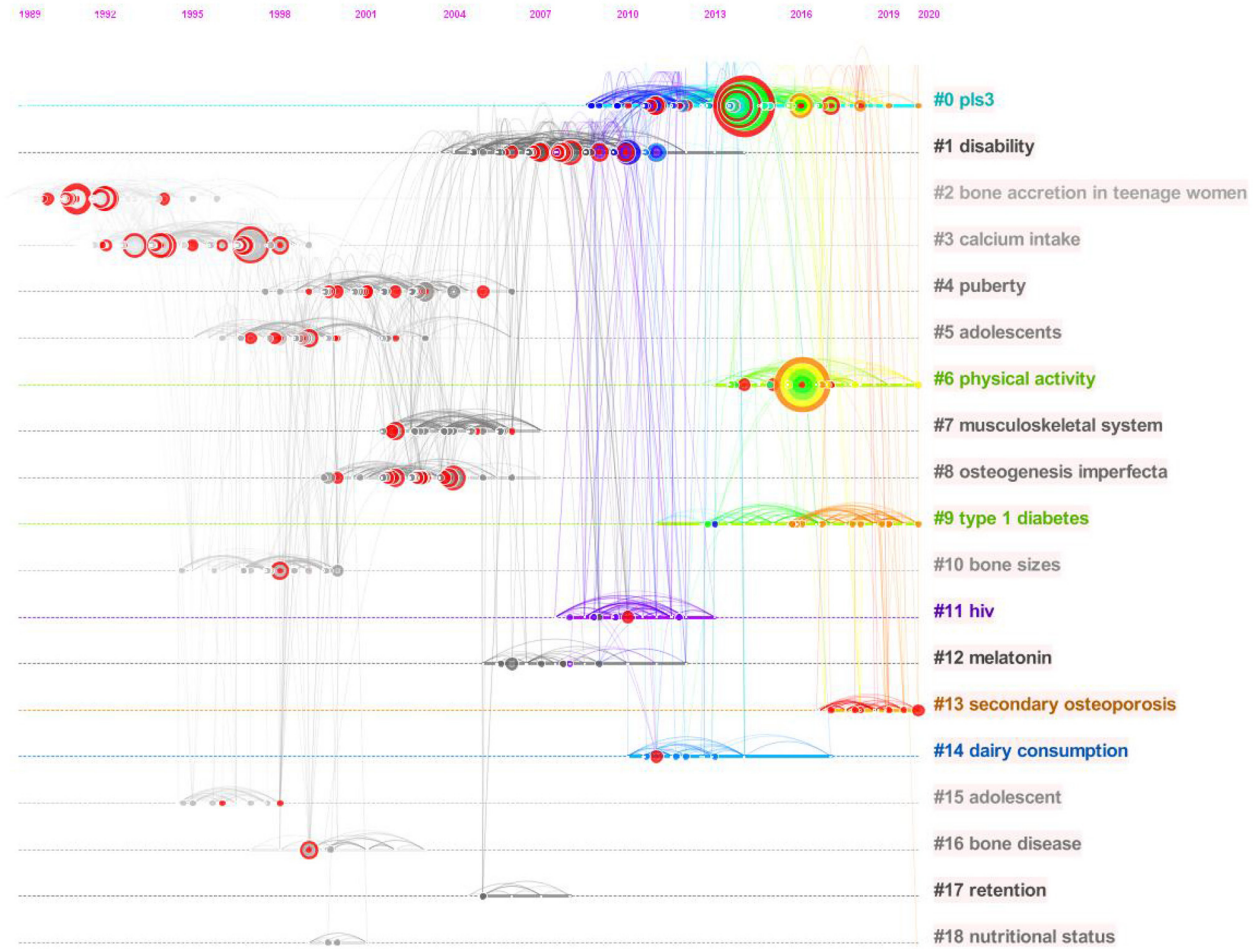
CiteSpace visualization map of timeline view of reference co-citation analysis.

**Figure 9:**
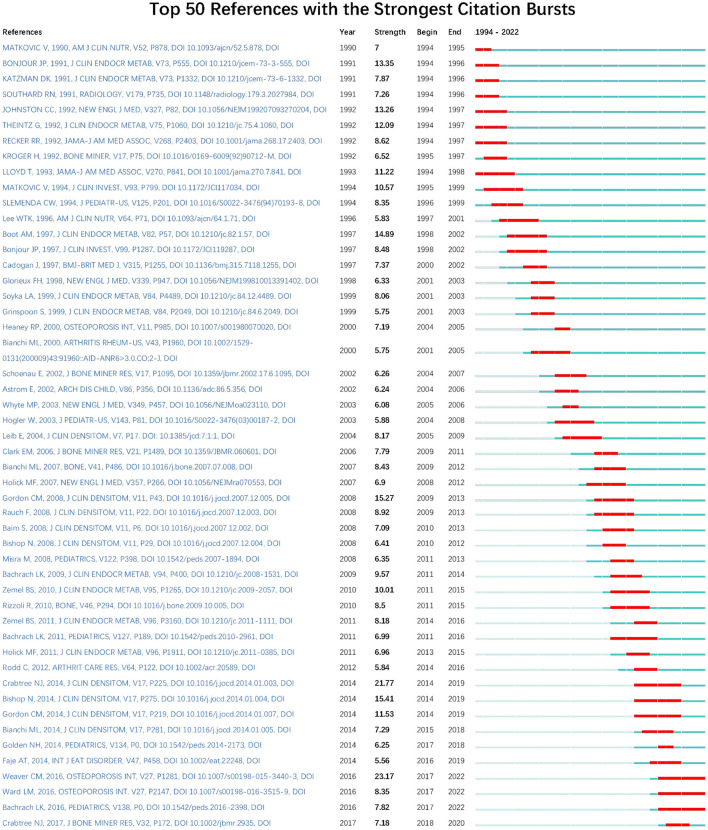
Top 50 references with the most robust citation bursts. The bars in red stand for a burst period for the references.

Table 2 summarizes the characteristics of the top 10 most cited papers within the AOP study, which have more than 300 citations, primarily concentrated in publications from around 1995 and 2008. In addition, Figure 8 shows a timeline view of the CiteSpace-generated literature co-citation network analysis, where all nodes representing citations can be grouped into 19 specific clusters. Clusters are grouped and ordered by the number of co-citations from 1989 to 2020. The time scale arranged horizontally at the top and the circle size suggests the amount of research on the subject. The first cluster is “#0pls3,” “#0pls3,” followed by “#1 disability” and “#2 bone accretion in teenage women.” Figure 9 shows the top 50 most situationally explosive papers identified through CiteSpace, with seven highlighting a duration of up to 5 years. Moreover, the most powerful citation explosion starting in 2017 came from the paper published by Weaver CM and colleagues in 2016, followed by the report by Crabtree et al.

**Table 2:** Characteristics of top 10 most-cited kinds of literature on AOP.

**Title**	**Journal**	**Author**	**Year**	**Citations**
Prevalence of vitamin D deficiency among healthy adolescents.	Arch Pediat Adol Med	Gordon CM	2004	583
Growth hormone, insulin-like growth factors, and the skeleton.	Endocr Rev	Giustina A	2008	575
Milk intake and bone mineral acquisition in adolescent girls: randomized, controlled intervention trial.	BMJ-Brit Med J	Cadogan J	1997	429
Calcium-enriched foods and bone mass growth in prepubertal girls: a randomized, double-blind, placebo-controlled trial.	J Clin Invest	Bonjour JP	1997	394
Jumping improves hip and lumbar spine bone mass in prepubescent children: A randomized controlled trial.	J Bone Miner Res	Fuchs RK	2001	385
American College of Sports Medicine position stand. The Female Athlete Triad.	Med Sci Sport Exer	Otis CL	1997	351
Weight-bearing exercise and bone mineral accrual in children and adolescents: A review of controlled trials.	Bone	Hind K	2007	327
The effects of estrogen administration on trabecular bone loss in young women with anorexia nervosa.	J Clin Endocr Metab	Klibanski A	1995	316
Low-magnitude mechanical loading is osteogenic in children with disabling conditions.	J Bone Miner Res	Ward K	2004	304
Childhood growth, physical activity, and peak bone mass in women	J Bone Miner Res	Cooper C	1995	300

## Discussion

Research on osteoporosis in adolescents has been increasing yearly in recent decades. Following the first peak in 2007, related research has stabilized mainly at a high level of enthusiasm since 2010. According to statistics, as many as half of boys and one-third of girls in healthy children will have a fracture by age 18, and one-fifth of children will have two or more fractures (10). Among them, the incidence of forearm fractures in children is as high as 33.33–50% (11). Seeman (12) believes that the risk of fracture and developing osteoporosis before and during adolescence is higher than after adolescence. The high risk of fracture increased the interest in this area. It is foreseeable that the literature in this area will continue to grow. It is worth noting that, in addition to the United States, Canada, and other countries that have historically produced a steady output of research results in areas related to osteoporosis in adolescents, Figure 3A shows that the research output of China (65; 733) has increased significantly in recent years, indicating that China is paying more and more attention to the progress of research in this area. The Children's Hospital and the UniChildren'sf Helsinki were the most prominent performers among the research institutions. Researchers can follow these countries and institutions to obtain timely information about research in this field.

The institutional cooperation network found that the institutions in Europe and the United States communicate more frequently. The number of Chinese patients is significant, but cooperation between national regions and institutions is still lacking. Immediately after, in terms of financial support, seven of the top 10 sources are from the United States, which shows its strong economic base and the importance it places on AOP research.

Regarding journal load, OSTEOPOROSIS INTERNATIONAL published the highest number of articles/reviews. The journal with the highest impact factor is JOURNAL OF BONE AND MINERALS RESEARCH. In addition, the JOURNAL OF PEDIATRICS impact factor increased significantly and exceeded six points in 2021. The dynamic changes in these journals should be noted in future studies. The biplot overlay analysis helps to understand the hot directions of research in different disciplines. As can be seen from the Figure 5C, there are three main citation paths in the biplot (green trace). Most of the papers in the AOP area are published in MEDICINE, MEDICAL, and CLINICAL; most of the documents are published in MOLECULAR, BIOLOGY, GENETICS, HEALTH, NURSING MEDICINE, SPORTS, REHABILITATION related journals were cited. In other words, early researchers focused more on clinical research; based on their predecessors, recent researchers have explored the pathogenesis of AOP from molecular, genetic, and biological aspects and concentrated on the rehabilitation and care of children with the disease.

In addition, three authors, Rauch F., Catherine M. Gordon, and Francis H. Glorieux, are recommended in the field of AOP. They are not only the first to join the AOP research field but also remain on the research front line. A critical study was published in 2000 by Rauch et al. (13). To investigate the pathogenesis of I.J.; they compared iliac bone biopsies of children with idiopathic juvenile osteoporosis, children with osteogenesis imperfecta, and healthy children. This study mentioned that 2-fold dysfunction of cancellous bone formation increased the risk of fracture in children with I.J. In 2002, Rauch and Glorieux et al. demonstrated for the first time histologically the effect of pamidophosphate in increasing bone mineral density in children with osteogenesis imperfecta (9). A recent study by Gordon et al. confirms the association of Crohn's disease with bone marrow obesity (14).

Among the literature citation networks, an original article entitled “Prevalence of vitamin D deficiency among healthy adolescents,” published in ARCH PEDIATR ADOL MED, was the most cited article in this field. This article investigates the prevalence of vitamin D deficiency in adolescents and highlights the importance of vitamin D for calcium absorption, bone growth, and augmentation during childhood and adolescence (15). The second most cited paper, by Giustina et al., describes the mechanism of action of the growth hormone (G.H.) and insulin-like growth factor-I (IGF-I) axes in bone and details the process by which abnormal levels of both lead to osteoporosis *in vivo* (16). The article with the third highest number of citations was published by Cadogan et al. This study concluded that increased milk intake significantly increased bone mineral acquisition and facilitated peak bone mass in adolescent girls without affecting bone turnover (17). Most of the top 10 articles are related to lifestyle adjustments such as diet and exercise. It is worth noting that three of them are from the journal J BONE MINER RES (18–20). “The Effects of Estrogen Administration on Trabecular Bone Loss in Young Women with Anorexia The Nervosa” prospectively used estrogen replacement therapy in young women with anorexia Nervosa and observed changes in bone mineral density (21). In addition, three papers (22–24) published by Weaver et al., Ward et al., and Bachrach et al. in 2016 are still in the hot state. Ward et al. reviewed the manifestations and risk factors associated with osteoporosis in children and recommended managing and preventing osteoporosis in adolescents. Bachrach et al. summarized methods for measuring bone mineral density in children and concluded that spinal bone mineral content (BMC) and BMD could be measured; whole-body measurements can be performed only in those over three y age. According to Weaver et al., lifestyle choices influence 20–40% of peak bone mass in adults, which is still emphasized by the latest official statement from Belgium (25).

High-frequency keywords are often used to generate co-occurrence network graphs to identify primary and hot topics in a research area for visualization of literature studies. In this study, we extracted 1,622 keywords from 1,199 articles. Through manual re-screening and inductive analysis, keywords that appeared more than five times were selected and classified into the following four groups. (1) Study the factors influencing and pathogenesis of AOP, such as calcium intake, peak bone mass, obesity, growth hormone, vitamin D, body composition, bone metabolism, and bone turnover. All of these may lead to changes in the cellular composition of bone tissue. In general, bone fragility is thought to be caused by loss of bone mass or defects in the composition or mineralization of the bone matrix (26). To date, the exploration of the pathogenesis of AOP is still active in the scientific planning of many investigators. (2) Research screening tests to assist in AOP diagnosis, including dual-energy x-ray absorptiometry, peripheral quantitative computed tomography, quantitative ultrasound, broadband ultrasound attenuation. With the development of the times, detection methods have become more and more diversified, and the research process never stops. (3) Research on the treatment of AOP, mainly exercise, lifestyle, bi sphosphonate, bisphosphonates, pamidronate, and zoledronic acid. Lifestyle modifications such as diet and exercise are always fundamental. Following the launch of injectable anti-osteoporosis drugs, the clinical use of teriparatide, denosumab, zoledronic acid, and other drugs, as well as the association between various systemic diseases affecting bone metabolism (especially the endocrine system) and the development of osteoporosis in adolescents have become the frontier of research in the field related to AOP and are still the high focus of clinical trials worldwide (27–30). (4) Research on AOP-related diseases, including but not limited to anorexia nervosa, osteogenesis imperfecta, cerebral palsy, inflammatory bowel disease, type 1 diabetes. Understanding the pathogenesis of these diseases in question will not only improve medical knowledge of the diseases but may also open the door to drug development for AOP.

## Limitations

Firstly, the dataset we included only includes data from the WoSSC database, which may leave out some relevant studies from other large databases. However, as the most used dataset in the field of bibliometrics, WoSSC also has sufficient data to illustrate the current state of the AOP research field. Secondly, we have excluded non-English language literature and therefore ignored the contribution to the field from some of the regions that use non-English languages. Third, as the database is constantly updated, the impact of recently published papers on the field may be underestimated.

## Conclusions

By using bibliometric and visual analysis, global research trends in AOP were identified in this study, in terms of number of publications, contributing countries, institutions, journals, authors, and references. For 29 years, the number of published studies on osteoporosis in adolescents has generally been on the rise, and the United States is leading the way in this field. The international cooperation map of the relevant countries/regions shows that the closest cooperation with the United States is in Canada and Italy. Children's Hospital and Professor Rauch F were the most prolific institutions and the most influential authors, respectively. OSTEOPOROSIS INTERNATIONAL is the most popular journal about AOP. Keywords co-occurrence analysis identified four clusters: etiological studies, AOP diagnostic studies, therapeutic studies, and related disease studies. In summary, the results of this study can provide practical resources for scholars to understand the current situation and trend of AOP research and provide references and suggestions for future related research.

## Data availability statement

All data generated and analyzed in this study is included in this work and available from the corresponding author upon reasonable request.

## Author contributions

DL, JO, and YZ designed the study. DL, YY, and ZL collected the data. The data was examined and the article was written by DL and JO. The draft of the paper was edited and polished by DL, JO, YZ, and LH. The article's submission was reviewed and approved by all authors.

## References

[1] The North American Menopause Society. Management of osteoporosis in postmenopausal women: The 2021 position statement of The North American Menopause Society. Menopause. (2021) 28:973–97. 10.1097/GME.0000000000001831 34448749

[2] Disser NP De Micheli AJ Schonk MM Konnaris MA Piacentini AN Edon DL. Musculoskeletal consequences of COVID-19. J Bone Joint Surg Am. (2020) 102:1197–204. 10.2106/JBJS.20.00847 32675661 PMC7508274

[3] Rouleau C Malorie M Collet C Porquet-Bordes V Gennero I Eddiry S. Diagnostic yield of bone fragility gene panel sequencing in children and young adults referred for idiopathic primary osteoporosis at a single regional reference centre. Bone Rep. (2022) 16:101176. 10.1016/j.bonr.2022.101176 35252483 PMC8892094

[4] Hou JW Wang TR. Idiopathic juvenile osteoporosis: Five-year case follow-up. J Formos Med Assoc. (1995) 94:277–80. 7613264

[5] Melchior R Zabel B Spranger J Schumacher R. Effective parenteral clodronate treatment of a child with severe juvenile idiopathic osteoporosis. Eur J Pediatr. (2005) 164:22–7. 10.1007/s00431-004-1541-7 15517381

[6] Kulkarni ML Keshavamurthy KS. Juvenile idiopathic osteoporosis. Indian Pediatr. (2004) 41:737–40. 15297692

[7] Chen C. Searching for intellectual turning points: Progressive knowledge domain visualization. Proc Natl Acad Sci USA. (2004) 101:5303–10. 10.1073/pnas.0307513100 14724295 PMC387312

[8] Jiang B Feng C Li C Tu C Li Z. A bibliometric and visualization analysis of glucocorticoid-induced osteoporosis research from 2012 to 2021. Front Endocrinol. (2022) 13:961471. 10.3389/fendo.2022.961471 35992120 PMC9388768

[9] Rauch F Travers R Plotkin H Glorieux FH. The effects of intravenous pamidronate on the bone tissue of children and adolescents with osteogenesis imperfecta. J Clin Invest. (2002) 110:1293–9. 10.1172/JCI0215952 12417568 PMC151613

[10] Rauch F Plotkin H DiMeglio L Engelbert RH Henderson RC Munns C. Fracture prediction and the definition of osteoporosis in children and adolescents: The ISCD 2007 Pediatric Official Positions. J Clin Densitom. (2008) 11:22–8. 10.1016/j.jocd.2007.12.003 18442750

[11] Bishop N Arundel P Clark E Dimitri P Farr J Jones G. Fracture prediction and the definition of osteoporosis in children and adolescents: The ISCD 2013 Pediatric Official Positions. J Clin Densitom. (2014) 17:275–80. 10.1016/j.jocd.2014.01.004 24631254

[12] Seeman E. Pathogenesis of bone fragility in women and men. Lancet. (2002) 359:1841–50. 10.1016/S0140-6736(02)08706-8 12044392

[13] Rauch F Travers R Norman ME Taylor A Parfitt AM Glorieux FH. Deficient bone formation in idiopathic juvenile osteoporosis: A histomorphometric study of cancellous iliac bone. J Bone Miner Res. (2000) 15:957–63. 10.1359/jbmr.2000.15.5.957 10804027

[14] Gordon RJ Pappa HM Vajapeyam S Mulkern R Ecklund K Snapper SB. Bone marrow adiposity in pediatric Crohn's disease. Bone. (2022) 162:116453. 10.1016/j.bone.2022.116453 35667602

[15] Gordon CM DePeter KC Feldman HA Grace E Emans SJ. Prevalence of vitamin D deficiency among healthy adolescents. Arch Pediatr Adolesc Med. (2004) 158:531–7. 10.1001/archpedi.158.6.531 15184215

[16] Giustina A Mazziotti G Canalis E. Growth hormone, insulin-like growth factors, and the skeleton. Endocr Rev. (2008) 29:535–59. 10.1210/er.2007-0036 18436706 PMC2726838

[17] Cadogan J Eastell R Jones N Barker ME. Milk intake and bone mineral acquisition in adolescent girls: Randomised, controlled intervention trial. Br Med J. (1997) 315:1255–60. 10.1136/bmj.315.7118.1255 9390050 PMC2127785

[18] Fuchs RK Bauer JJ Snow CM. Jumping improves hip and lumbar spine bone mass in prepubescent children: A randomized controlled trial. J Bone Miner Res. (2001) 16:148–56. 10.1359/jbmr.2001.16.1.148 11149479

[19] Ward K Alsop C Caulton J Rubin C Adams J Mughal Z. Low magnitude mechanical loading is osteogenic in children with disabling conditions. J Bone Miner Res. (2004) 19:360–9. 10.1359/JBMR.040129 15040823

[20] Cooper C Cawley M Bhalla A Egger P Ring F Morton L. Childhood growth, physical activity, and peak bone mass in women. J Bone Miner Res. (1995) 10:940–7. 10.1002/jbmr.5650100615 7572318

[21] Klibanski A Biller BM Schoenfeld DA Herzog DB Saxe VC. The effects of estrogen administration on trabecular bone loss in young women with anorexia nervosa. J Clin Endocrinol Metab. (1995) 80:898–904. 10.1210/jcem.80.3.7883849 7883849

[22] Bachrach LK Gordon CM. Bone densitometry in children and adolescents. Pediatrics. (2016) 138:e20162398. 10.1542/peds.2016-2398 27669735

[23] Ward LM Konji VN Ma J. The management of osteoporosis in children. Osteoporos Int. (2016) 27:2147–79. 10.1007/s00198-016-3515-9 27125514

[24] Weaver CM Gordon CM Janz KF Kalkwarf HJ Lappe JM Lewis R. The National Osteoporosis Foundation's position statement on peak bone mass development and lifestyle factors: A systematic review and implementation recommendations. Osteoporos Int. (2016) 27:1281–386. 10.1007/s00198-015-3440-3 26856587 PMC4791473

[25] Laurent MR Goemaere S Verroken C Bergmann P Body JJ Bruyère O. Prevention and treatment of glucocorticoid-induced osteoporosis in adults: Consensus recommendations from the belgian bone club. Front Endocrinol. (2022) 13:908727. 10.3389/fendo.2022.908727 35757436 PMC9219603

[26] El-Gazzar A Högler W. Mechanisms of bone fragility: From osteogenesis imperfecta to secondary osteoporosis. Int J Mol Sci. (2021) 22:625. 10.3390/ijms22020625 33435159 PMC7826666

[27] Gagliardi I Celico M Gamberini MR. Efficacy and safety of teriparatide in beta-thalassemia major associated osteoporosis: A real-life experience. Calcif Tissue Int. (2022) 22:3. 10.1007/s00223-022-00963-3 35243531 PMC9232424

[28] Shane E Shiau S Recker RR. Denosumab after teriparatide in premenopausal women with idiopathic osteoporosis. J Clin Endocrinol Metab. (2022) 107:e1528–40. 10.1210/clinem/dgab850 34849989 PMC9122662

[29] Ward LM Choudhury A Alos N. Zoledronic acid vs. placebo in pediatric glucocorticoid-induced osteoporosis: A randomized, double-blind, phase 3 trial. J Clin Endocrinol Metab. (2021) 106:e5222–35. 10.1210/clinem/dgab458 34228102

[30] Barr RD Inglis D Athale U. Bone health in long-term survivors of pediatric acute lymphoblastic leukemia. An assessment by peripheral quantitative computed tomography. Pediatr Blood Cancer. (2021) 68:e29218. 10.1002/pbc.29218 34264535

